# TAILOR: Transgene Activation and Inactivation Using Lox and Rox in Zebrafish

**DOI:** 10.1371/journal.pone.0085218

**Published:** 2013-12-31

**Authors:** Joon Tae Park, Steven D. Leach

**Affiliations:** 1 Department of Surgery, Johns Hopkins Medical Institutions, Baltimore, Maryland, United States of America; 2 McKusick-Nathans Institute of Genetic Medicine, Johns Hopkins Medical Institutions, Baltimore, Maryland, United States of America; National University of Singapore, Singapore

## Abstract

The ability to achieve precisely tailored activation and inactivation of gene expression represents a critical utility for vertebrate model organisms. In this regard, *Cre* and other site-specific DNA recombinases have come to play a central role in achieving temporally regulated and cell type-specific genetic manipulation. In zebrafish, both *Cre* and *Flp* recombinases have been applied for inducible activation, inactivation and inversion of inserted genomic elements. Here we describe the addition of *Dre*, a heterospecific *Cre*-related site-specific recombinase, to the zebrafish genomic toolbox. Combining *Dre*-based recombination in zebrafish with established *Cre/lox* technology, we have established an effective strategy for transgene activation and inactivation using *lox* and *rox* (TAILOR). Using stable transgenic lines expressing tamoxifen-inducible *CreER^T2^* and RU486-inducible *DrePR* fusions, we demonstrate that *Cre* and *Dre* retain non-overlapping specificities for their respective *lox* and *rox* target sites in larval zebrafish, and that their combinatorial and sequential activation can achieve precisely timed transgene activation and inactivation. In addition to TAILOR, the successful application of *Dre/rox* technology in zebrafish will facilitate a variety of additional downstream genetic applications, including sequential lineage labeling, complex genomic rearrangements and the precise temporal and spatial control of gene expression through the intersection of partially overlapping promoter activities.

## Introduction

Site-specific DNA recombinases have become critical components of genome manipulation strategies in vertebrates. In zebrafish, both the *Cre/lox* and *Flp/frt* systems have been utilized for a variety of applications, including transgene activation, transgene excision, and transgene inversion [1]–[16]. Recently, an additional *Cre*-like site-specific recombinase known as *Dre* was identified by sequencing homologous genomic regions of D6, a transducing bacteriophage related to the P1 phage from which *Cre* was isolated [17]. Like *Cre*, *Dre* is a site-specific tyrosine recombinase capable of catalyzing both excision and integration events, with no requirement for other phage-encoded or bacterial proteins. While *Cre* displays specificity for *lox* target sites, *Dre* recognizes a different target sequence known as *rox*. While *lox* and *rox* sites are similar in size (34 bp vs. 32 bp), sequence (21 nucleotides in common) and structure (inverted repeats flanking an asymmetric spacer), *Cre* has no ability to recombine *rox* sites and *Dre* similarly has no activity at *lox* sites, either in bacteria or in mammalian cells [18], [19].

Based on the recent successful application of the *Dre/rox* system in mice [18], we sought to determine its activity in zebrafish embryos, with an emphasis on developing new techniques for sequential activation and inactivation of inserted transgenes.

## Materials and Methods

### Transgenesis

All experiments involving zebrafish were approved by the Johns Hopkins University Institutional Animal Care and Use Committee. Fish were raised and maintained under standard laboratory conditions. The following strains were established and/or utilized: *Tg(ubb:Dre;cryaa:Venus)* (herein *ubb-Dre*), *Tg(ubb:Cre;cryaa:Venus)* (herein *ubb-Cre*), *Tg(ubb:lox-Nuc-mCherry-stop-lox-eGFP)* (herein *Lox-Nuc-mCherry-Lox-eGFP*), *Tg(ubb:rox-Nuc-mCherry-stop-rox-eGFP)* (herein *Rox-Nuc-mCherry-Rox-eGFP*), *Tg(ubb:DrePR;cryaa:eCFP)* (herein *ubb-DrePR*), *Tg(ubb:CreER^T2^;cmlc2:eGFP)* (herein *CreER^T2^*) [20], *Tg(ubb:lox-stop-lox-rox-nuc-mCherry-stop-rox-eGFP;cryaa:mCherry)* (herein *LSL-Rox-Nuc-mCherry-Rox-eGFP*). *Dre* and *DrePR* cDNAs were kindly provided by Dr. Francis Stewart. All new transgenic lines were generated using the backbone of T2KXIGΔIN [21], as previously described [22]. Larvae were anaesthetized in 0.16% tricaine (3-aminobenzoic acid ethylester, A-5040, Sigma, pH 7.0). Adult zebrafish were euthanized by induction of tricaine anesthesia followed by placement in an ice bath, consistent with recommendations of the Panel on Euthanasia of the American Veterinary Association.

### 4-OHT Treatment for *CreER^T2^* Induction and RU486 Treatment for *DrePR* Induction

4-Hydroxytamoxifen (4-OHT, H7904; Sigma, St Louis, MO, USA) and RU486 (H110-01; Invitrogen) were dissolved in ethanol at a final stock concentration of 10 mM and kept in single-use aliquots in the dark at –20°C. To induce *Cre* activity in *CreER^T2^*-expressing embryos, 25–30 stage-matched embryos were incubated with E3 medium freshly mixed with 5 µM 4-OHT. To induce *Dre* activity in *DrePR*-expressing embryos, 25–30 stage-matched embryos were incubated with E3 medium freshly mixed with 4 µM RU486. The treated embryos were immediately put into a closed and dark 28.5°C incubator for 24 hrs. The embryos were subsequently placed in a fresh E3 medium and grown as described previously.

### Tissue Dissection, Confocal Microscopy, and Cell Counting

Tissue dissection and confocal microscopy were performed as described previously [22]. To quantify the efficiency of *DrePR* recombination in a RU486-dose dependent manner, 25–30 stage-matched embryos (n = 5) were treated with E3 medium freshly mixed with 0 µM, 1 µM, 2 µM, 4 µM, and 10 µM of RU486 between 24 and 48 hpf. Two days later, 4 dpf the larval zebrafish were fixed overnight in 4% PFA and the intestine was dissected and mounted in the mounting media (DAKO, S3023). To quantify the efficiency of sequential *CreER^T2^*- and *DrePR*-mediated recombination, larval zebrafish (n = 5) were treated sequentially with 4-OHT at 24 hpf and RU486 at 48 hpf, and fixed overnight in 4% PFA at 7 dpf. Following dissection, sections of intestine, liver, and pancreas were prepared. The total number of DAPI-labeled cells also labeled by either mCherry or eGFP was counted, with a minimum of 400 cells counted for each tissue.

## Results

### *Dre* Effectively Recombines *Rox* Sites but not *Lox* Sites in Larval Zebrafish

**Figure 1:**
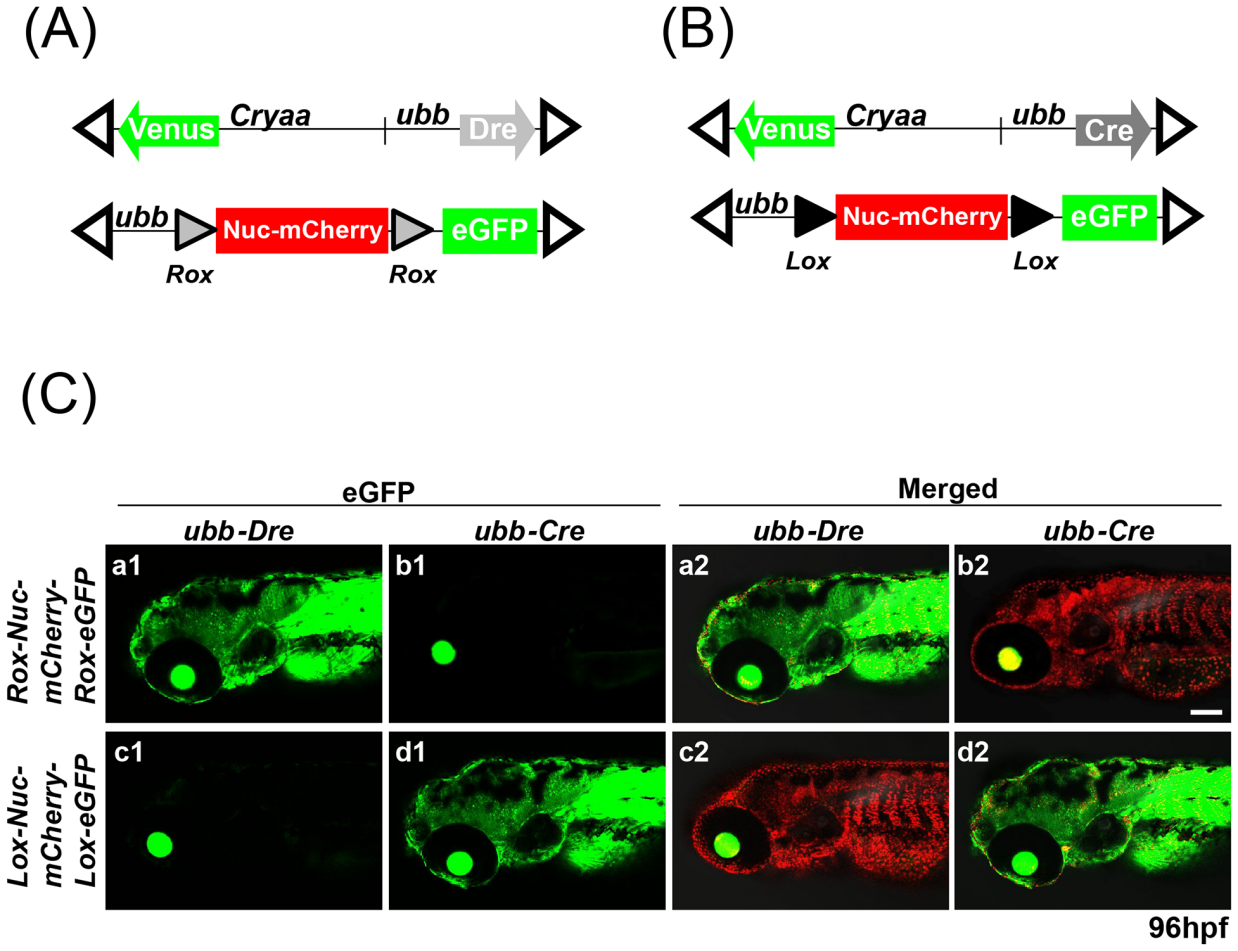
Heterospecific recombination of *rox* and *lox* sites by *Dre* and *Cre* in zebrafish embryos. (**A, B**) Schematic of *ubb-Dre* (A) and *ubb-Cre* (B) driver lines and corresponding *Rox-Nuc-mCherrys-Rox* and *Lox-Nuc-mCherry-Lox* reporters. Additional *cryaa:Venus* cassette facilitates identification of transgene-expressing embryos. Open triangles indicate Tol2 arms. (**C**) Images from double transgenic embryos produced by indicated crosses of *ubb-Dre* and *ubb-Cre* driver lines with either *Rox-Nuc-mCherry-Rox-eGFP* or *Lox-Nuc-mCherry-Lox-eGFP* reporter lines. Activation of eGFP confirms *Dre*-specific recombination of *Rox-Nuc-mCherry-Rox* reporter and *Cre*-specific recombination of *Lox-Nuc-mCherry-Lox* reporter. Scale bar: 200 µm.

As an initial evaluation of *Dre*-based recombination in zebrafish, we utilized standard Tol2-mediated transgenesis to establish *Dre*-driver and a *Dre*-responder lines in which relevant elements are expressed under the control of *zebrafish ubiquitin b* (*ubb*) promoter (Fig. 1A). The *ubb* promoter drives transgene expression ubiquitously in the vast majority of cell types through all stages of zebrafish development, beginning at the mid-blastula transition [20]. The *Dre* driver transgene (*ubb:Dre;cryaa:Venus,* subsequently referred to as *ubb-Dre*) also incorporated a *crystalline aa:Venus* cassette [23], with resulting Venus fluorescence in the eye facilitating the identification of transgene-expressing embryos. To establish stable transgenic lines, we selected two independent male F1 founders whose F2 progeny displayed a ∼50% incidence of Venus eye fluorescence, suggesting a single *ubb-Dre* transgene integration. The *Dre* responder line contained a *Nuc-mCherry-stop* cassette flanked by 32 bp *rox* sites and followed by eGFP (*ubb-rox-Nuc-mCherry-stop-rox-eGFP,* subsequently referred to as *Rox-Nuc-mCherry-Rox-eGFP*). Stable *Dre* responder lines were selected based on ubiquitous expression of *ubb* promoter-driven nuclear mCherry, evident shortly following the mid-blastula transition.

To confirm non-overlapping specificities for the *Dre/rox* and *Cre/lox* systems in larval zebrafish, we also established *Cre*-driver and *Cre*-responder lines under the control of the *ubb* promoter (Fig. 1B). As in the case of *ubb-Dre, the ubb-Cre* transgene also contained *crystalline aa:Venus* cassette. We established three independent male F1 founders carrying a single *ubb-Cre* transgene integration. A similar *Cre* responder line was also established, with a *Nuc-mCherry-stop* cassette flanked by *lox* sites and followed by eGFP (*ubb-lox-Nuc-mCherry-stop-lox-eGFP,* subsequently referred to as *Lox-Nuc-mCherry-Lox-eGFP*) (Fig. 1B).

To initially determine whether *Dre* was active in larval zebrafish, we crossed male *ubb-Dre* fish to female *Rox-Nuc-mCherry-Rox-eGFP* fish. These experiments confirmed efficient *Dre*-based deletion of the *Rox-Nuc-mCherry-Rox* cassette, as evidenced by activation of eGFP expression (Fig. 1C a1 and a2). To further confirm that *Dre* does not recognize related *lox* sites, we also crossed *ubb-Dre* males with *Lox-Nuc-mCherry-Lox-eGFP* females. In contrast to *Rox-Nuc-mCherry-Rox-eGFP*, no activation of *eGFP* expression was observed (Fig. 1C c1 and c2). Extending these studies to the evaluation of *Cre* activity, we similarly crossed *ubb-Cre* males to both *Lox-Nuc-mCherry-Lox-eGFP* and *Rox-Nuc-mCherry-Rox-eGFP* females. As expected, *Cre* effectively activated the conditional *Lox-Nuc-mCherry-Lox-eGFP* allele (Fig. 1C d1 and d2), but showed no ability to activate eGFP expression in the *Rox-Nuc-mCherry-Rox-eGFP* line (Fig. 1C b1 and b2). Thus *Cre* and *Dre* behave as heterospecific DNA recombinases when expressed as stable transgenes in larval zebrafish.

### Induction of *DrePR* by RU486

**Figure 2:**
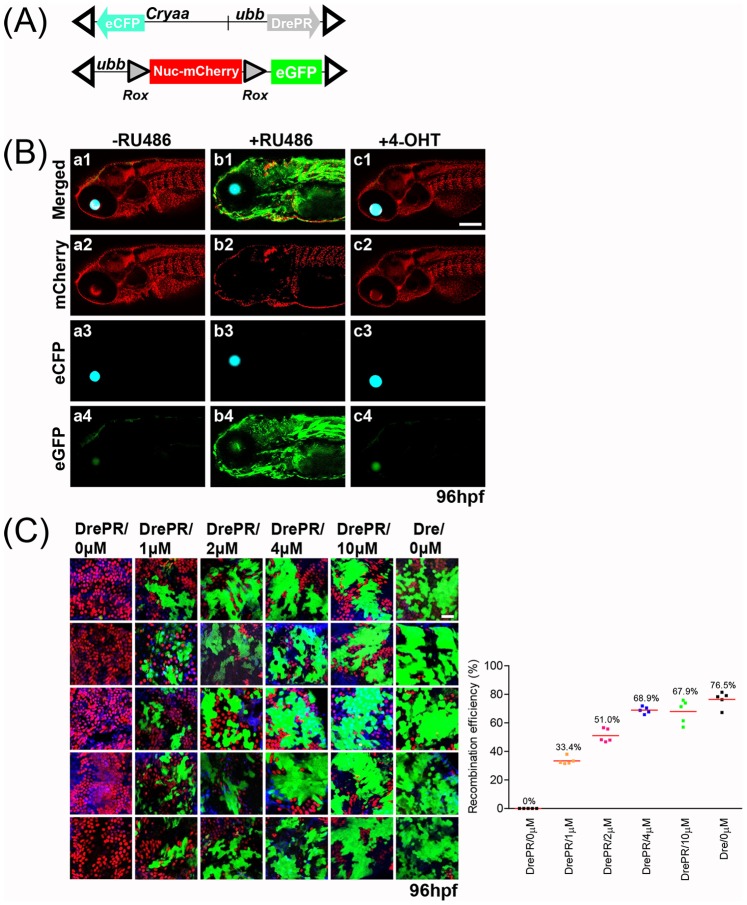
Induction of *DrePR* recombinase activity by RU486. (**A**) Schematic of *ubb-DrePR* driver line and *Rox-Nuc-mCherry-Rox* reporter. Additional *cryaa:eCFP* cassette facilitates identification of transgene-expressing embryos. Open triangles indicate Tol2 arms. (**B**) Tight control of *DrePR* recombinase activity by RU486. *ubb-DrePR*; *Rox-Nuc-mCherry-Rox-eGFP* embryos were treated with and without 4 µM RU486 between 24 and 48 hpf, and imaged at 96 hpf. No expression of eGFP is observed in untreated (-RU486) or tamoxifen (4-OHT)-treated embryos, while treatment with RU486 results in potent induction of eGFP expression indicating successful recombination of *Rox-Nuc-mCherry-Rox-eGFP* allele. (**C**) To quantify the efficiency of *Dre* recombination in *Dre*-expressing embryos and *DrePR* recombination in *DrePR*-expressing embryos, the intestine of larval zebrafish (4 dpf) were dissected following treatment with RU486 at the indicated concentration between 24–48 hpf. DAPI-labeled cells also labeled by either nucleus mCherry or cytoplasmic eGFP were counted. Maximal recombination frequency is achieved an an RU486 concentration of 4 µM, at a level comparable with Dre lacking the PR fusion. Scale bar: 25 µm.

In order to establish a temporally-inducible system for the regulation of *Dre* activity that would complement already established *CreER^T2^* lines, we adapted an expression construct for *DrePR* [18], in which *Dre* is fused to a progesterone receptor (*PR*) ligand binding domain engineered to be selectively responsive to the synthetic ligand RU486. First, we established a *DrePR*-driver line under control of the *ubb* promoter. To facilitate the identification of *DrePR*-expressing embryos, this transgene (*ubb:DrePR;cryaa:eCFP* subsequently referred to as *ubb-DrePR*) also incorporated a *crystalline aa:eCFP* cassette [23] in the opposite direction of the *ubb* promoter (Fig. 2A). To establish stable transgenic lines, we selected three independent male F1 founders whose F2 progeny displayed a ∼50% incidence of eCFP eye fluorescence, suggesting a single *ubb-DrePR* transgene integration. To characterize *ubb*-driven *DrePR* function, we crossed a male *ubb-DrePR* line to a female *Rox-Nuc-mCherry-Rox-eGFP* line. To induce *Dre* activity in *DrePR*-expressing embryos, 25–30 stage-matched embryos were incubated in the absence and presence of 4 µM RU486 for 24 hrs. No eGFP was expressed in *DrePR*-expressing embryos incubated in the absence of RU486 (Fig. 2B a1 and a4), but strong eGFP activation was observed in RU486-treated embryos (Fig. 2B b1 and b4). To test for any unanticipated cross-reactivity between 4-OHT and RU486, we also treated *DrePR*-expressing embryos with 5 µM 4-OHT, and observed no induction of eGFP expression (Fig. 2B c1 and c4).

Prior studies have suggested that *CrePR* fusions display less effective recombinase activity compared to native *Cre* [24], [25]. To check whether the *DrePR* fusion might be associated with a similar reduction in *Dre* recombinase activity, we crossed both *ubb-DrePR* and *ubb-Dre* males to *Rox-Nuc-mCherry-Rox-eGFP* females. In the case of *DrePR*, 25–30 stage-matched embryos (n = 5) were treated with E3 medium freshly mixed with RU486-dose dependent manner, 0 µM, 1 µM, 2 µM, 4 µM, and 10 µM. To quantify the efficiency of *Dre* recombination in *Dre*-expressing embryos and *DrePR* recombination in *DrePR*-expressing embryos, the intestine of larval zebrafish (4 dpf) was dissected and DAPI-labeled cells also labeled by either nucleus mCherry or cytoplasmic eGFP was counted. A dose-response curve revealed that the efficiency of RU486-induced DrePR activation became saturated at between 2 and 4 µM of RU486. No cytoplasmic eGFP-labeled cells were observd in *DrePR* embryos in the absence of RU486 (Fig. 2C), further confirming the absence of leaky DrePR activity. Furthermore, the maximal recombination activity of *DrePR* (68.9% at 4 µM of RU486) was similar to that of non-inducible *Dre* lacking the PR fusion (76.5%), indicating that C-terminal fusion of *Dre* with the *PR* does not interfere greatly with recombinase functionality (Fig. 2C). These data confirm tight and efficient temporal control of *DrePR* activity by RU486.

### Combinatorial Activation of *DrePR* and *CreER^T2^*

**Figure 3:**
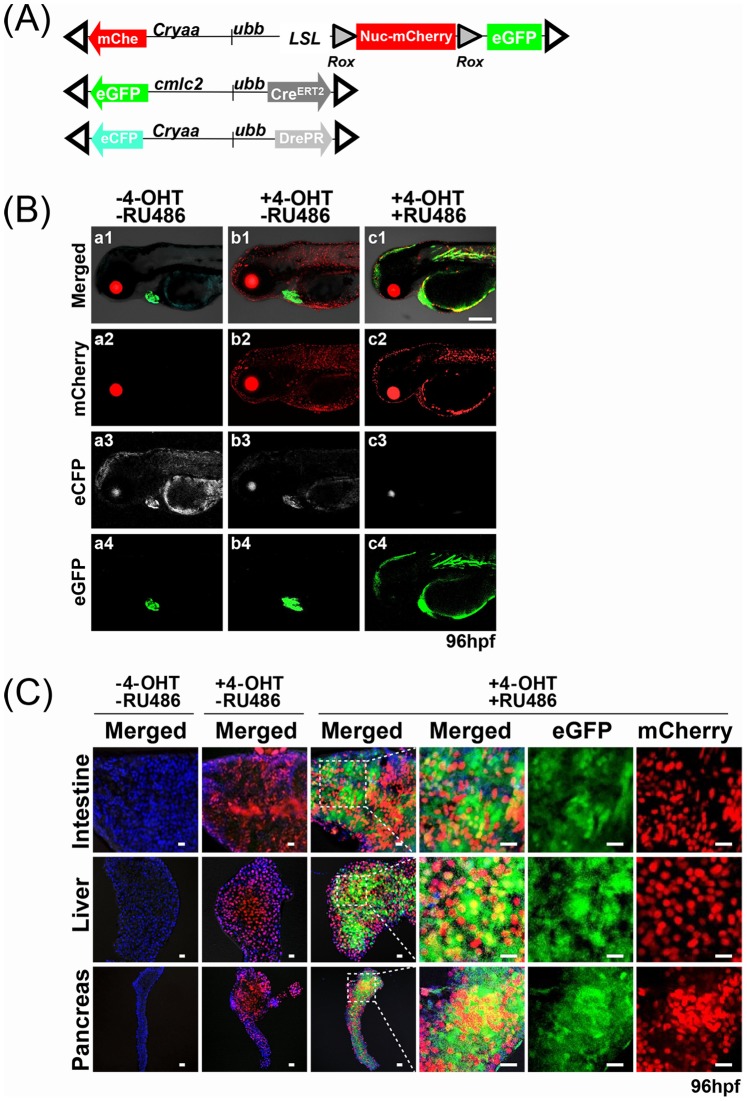
Combinatorial activation of *DrePR* and *CreER^T2^*. (**A**) Schematic of *ubb:lox-stop-lox-rox-Nuc-mCherry-stop-rox-eGFP* dual reporter for assessment of both *Cre*- and *Dre*-mediated recombination, along with *ubb-CreER^T2^* and *ubb-DrePR* driver lines. Ocular and cardiac fluorescence conveyed by additional *cryaa:mCherry*, *cmlc2:eGFP and cryaa:eCFP* cassettes facilitates identification of transgene-expressing embryos. Open triangles indicate Tol2 arms. (**B**) Triple transgenic fish were treated for 24 hrs with or without 4-OHT and RU486 as indicated, and imaged at 96 hpf. While untreated embryos showed no transgene-specific fluorescence besides that provided by the ocular mCherry, ocular eCFP and cardiac eGFP markers (Fig. 3B a1, a2, a3, and a4), embryos treated with only 4-OHT displayed widespread activation of nuc-mCherry, but no activation of eGFP (Fig. 3B b1, b2, b3, b4). In contrast, embryos simultaneously treated with both 4-OHT and RU486 displayed expression of both nuc-mCherry and eGFP (Fig. 3B c1, c2, c3, and c4). Scale bar: 200 µm. (**C**) Confocal imaging of dissected intestine, liver, and pancreas, confirming patterns of mCherry and eGFP expression observed in whole embryos. Following combined treatment with 4-OHT and RU486, a majority of cells in each tissue express either nuclear mCherry or cytoplasmic eGFP, with a smaller fraction of cells expressing both. Scale bar: 25 µm.

In order to further evaluate the combinatorial utilities of *DrePR* and *CreER^T2^*, we established an additional responder line incorporating fluorescent reporters of both *Dre* and *Cre* recombinase activity. To facilitate the identification of transgene expressing embryos, this dual reporter construct (*ubb:lox-Stop-lox-rox-Nuc-mCherry-stop-rox-eGFP;cryaa:mCherry,* subsequently referred to as *LSL-Rox-Nuc-mCherry-Rox-eGFP*) also incorporated a *crystalline aa:mCherry* cassette [23], placed in an opposite orientation relative to the *ubb* promoter. To generate a stable, temporally-regulated Cre driver line, we used a 4-OHT inducible *CreERT2* element under the control of *ubb* promoter [20]. This line (*ubb:CreER^T2^;cmlc2:eGFP,* subsequently referred to as *ubb-CreER^T2^*) also incorporated *cmlc2:eGFP* cassette to facilitate the identification of transgene-expressing embryos based upon cardiac expression of eGFP. Following the creation of double-transgenic *ubb-CreER^T2^*; *ubb-DrePR* fish, additional crosses were completed to assemble these *CreER^T2^* and *DrePR* driver alleles together with the *LSL-Rox-Nuc-mCherry-Rox-eGFP* reporter (Fig. 3A).

We then tested whether *CreER^T2^* and *DrePR* could be combinatorially applied for the inducible activation of a tandem “*LSL-Rox-Nuc-mCherry-Rox*” allele, as would be required if transgene activation was desired only in a highly selective subset of cells expressing both Cre and Dre by virtue of the intersectional expression of two different promoters [26]–[28]. We treated 25–30 stage-matched *LSL-Rox-Nuc-mCherry-Rox-eGFP; ubb-CreER^T2^; ubb-DrePR* triple transgenic embryos with either 4-OHT alone or the combination of both 4-OHT and RU486, applied simultaneously. While untreated embryos showed no transgene-specific fluorescence besides that provided by the ocular mCherry, ocular eCFP and cardiac eGFP markers (Fig. 3B a1, a2, a3, and a4), embryos treated with only 4-OHT displayed widespread activation of nuc-mCherry, but no activation of eGFP (Fig. 3B b1, b2, b3, and b4). In contrast, embryos simultaneously treated with both 4-OHT and RU486 displayed expression of both nuc-mCherry and eGFP (Fig. 3B c1, c2, c3, and c4). To examine activation of nuc-mCherry and eGFP at the cellular level, we dissected internal organs (intestine, liver, and pancreas) for confocal imaging. These studies confirmed observations made in whole mount embryos, with a majority of cells in each tissue expressing either the nuclear mCherry or the cytoplasmic eGFP markers, and a smaller fraction of cells expressing both (Fig. 3C). Several mechanisms may underlie the mosaic expression patterns observed in embryos treated with both 4OHT and RU486. While cells expressing eGFP alone have obviously undergone recombination of both *lox*- and the *rox*-flanked elements, cells expressing only mCherry have presumably undergone effective 4-OHT induction of *CreER^T2^* but failed to achieve RU486 induction of *DrePR* activity at threshold levels required for excision of the *Rox-Nuc-mCherry-Rox* cassette. Cells expressing both mCherry and eGFP may have undergone sequential *CreER^T2^* and *DrePR*-mediated recombination, such that an interval of stable mCherry protein production precedes *DrePR*-mediated excision. Alternatively, expression of both mCherry and eGFP may reflect discordant recombination of more than one Tol2-based *LSL-Rox-Nuc-mCherry-Rox-eGFP* insert, in spite of our attempts to utilize only F1 founders passing a single *LSL-Rox-Nuc-mCherry-Rox-eGFP* allele.

### Sequential Transgene Activation and Inactivation using *Lox* and *Rox* (TAILOR)

**Figure 4:**
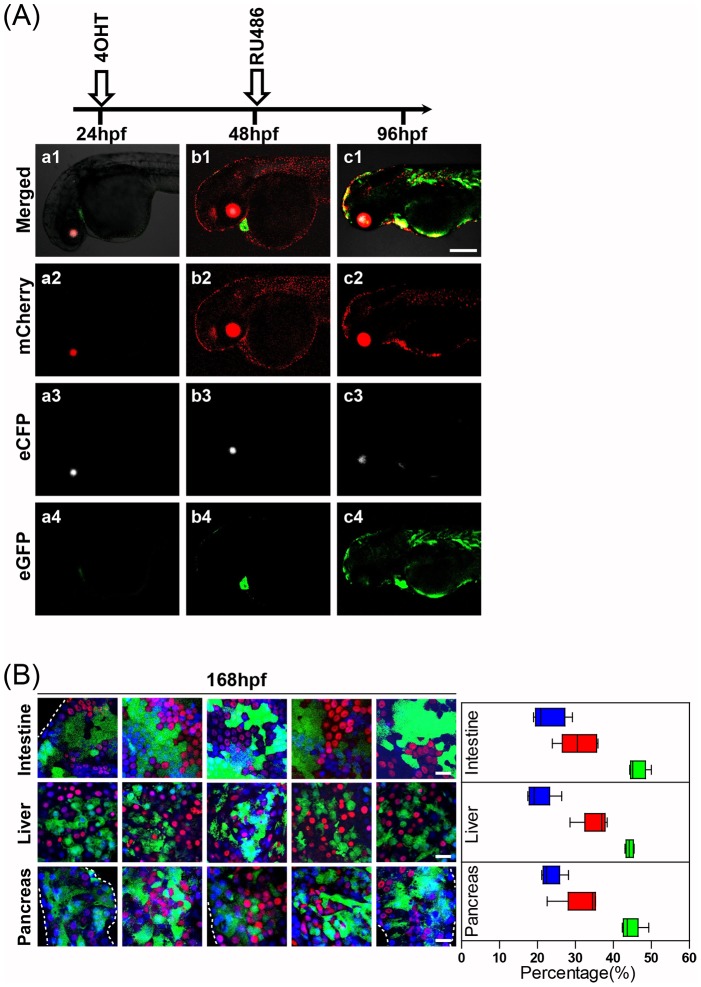
Sequential transgene activation and inactivation using *lox* and *rox* (TAILOR). (**A**) *ubb:lox-stop-lox-rox-Nuc-mCherry-stop-rox-eGFP; ubb-CreER^T2^; ubb-Dre* triple transgenic fish were subjected to treatment with 4-OHT beginning at 24 hpf, followed by removal and replacement with RU486 at 48 hpf. The untreated triple transgenic embryos (24 hpf) showed no transgene-specific fluorescence besides that provided by the ocular mCherry, ocular eCFP and cardiac eGFP markers (Fig. 4A a1, a2, a3, and a4). The effective induction of nuclear mCherry expression was observed following 24 hrs of 4-OHT treatment (Fig. 4A b1 and b2). Following staged treatment with RU486 initiated at 48 hpf and left in place for 24 hrs, effective activation of eGFP expression was observed (Fig. 4A c1 and c4). Scale bar: 200 µm. (**B**) Quantification of relative numbers of intestinal, liver and pancreatic cells expressing mCherry (red), eGFP (green) or neither (blue) at 7 days following sequential 4OHT and RU486 exposure as above. Note high fraction of cells undergoing sequential *CreER^T2^*-mediated activation and *DrePR*-mediated inactivation of mCherry expression, as indicated by eGFP expression. Scale bar: 25 µm.

In addition to simultaneous recombination of *lox* and *rox* sites for selected activation of transgenes in cells expressing both *CreER^T2^* and *DrePR*, many additional applications would be enabled by the staged induction of recombinase activity. Among these would be the sequential activation and inactivation of a specific transgene in a temporally-regulated, cell type-specific manner. We therefore conceived a strategy for transgene activation and inactivation using *lox* and *rox* **,** referred to as TAILOR. Using the same *LSL-Rox-Nuc-mCherry-Rox-eGFP; ubb-CreER^T2^; ubb-DrePR* triple transgenic embryos depicted in Fig. 3A, we sought to determine the feasibility and efficiency of tamoxifen-inducible *LSL-Rox-Nuc-mCherry-Rox* transgene activation, followed by RU486-inducible transgene deletion. For these studies, 24 hpf triple transgenic embryos were treated with 4-OHT for 24 hrs, after which 4-OHT was removed from the embryo medium and replaced with RU486. Individual embryos were serially imaged prior to (48 hpf) and after (96 hpf) treatment with RU486 (Fig. 4A).

The untreated triple transgenic embryos (24 hpf) showed no transgene-specific fluorescence besides that provided by the ocular mCherry, ocular eCFP and cardiac eGFP markers (Fig. 4A a1, a2, a3, and a4). The effective induction of nuclear mCherry expression was observed following 24 hrs of 4-OHT treatment (Fig. 4A b1 and b2). Nuc-mCherry signal was detected by immunofluorescence within 6 hrs of 4-OHT treatment, and signal was widely expressed in all regions of embryo following 24 hrs of 4-OHT treatment. Following staged treatment with RU486 initiated at 48 hpf and left in place for 24 hrs, effective activation of eGFP expression was observed (Fig. 4A c1 and c4). The activation of eGFP fluorescence was evident within 6 hrs of RU486 application, indicating the rapid excision of *rox*-flanked mCherry in at least a subset of larval cells.

Not unexpectedly, embryos treated sequentially with 4-OHT and RU486 and harvested at 96 hpf continued to have a fraction of cells displaying ongoing red nuclear fluorescence (Fig 4A.c.2), potentially reflecting both perdurant mCherry protein and/or mosaic DrePR activity. In order to more formally document sequential transgene activation and inactivation, as well as to calculate the efficiency of sequential *CreER^T2^*- and *DrePR*-based recombination, we delayed harvest until 7 dpf and examined patterns of mCherry and eGFP fluorescence in intestine, liver and pancreas. This allowed us to precisely determine the number of cells undergoing either no recombination, single recombination mediated by *CreER^T2^*, or double recombination mediated by the sequential activity of *CreER^T2^* and *DrePR*. Minimal variation in recombination frequencies was observed between these three tissues, with 20–25% of cells undergoing no recombination (blue DAPI fluorescence only), 30–40% of cells undergoing single recombination mediated by CreER^T2^ (red mCherry fluorescence only), and 40–50% of cells undergoing sequential transgene activation and inactivation mediated by both *CreER^T2^* and *DrePR* (green eGFP fluorescence only).

## Discussion

Together, these studies establish *Dre/rox*-based recombination as a new and effective method for temporally- and spatially-regulated genetic manipulation in zebrafish. In addition to this general utility, we present a new strategy for sequential activation and inactivation of inserted transgenes, referred to as TAILOR. Prior methods for temporally regulated transgene expression in zebrafish have included the use of thermally-inducible heat shock promoters [29], [30], the application of modified tetracycline-inducible systems [31], [32], and the development of hormone-regulated hybrid transcriptional activators [33]. More recently, DNA recombinases have been employed for temporal regulation of zebrafish transgene expression, and the power of combinatorial approaches utilizing multiple recombinases has become apparent [12], [13], [34].

The current addition of *Dre/rox* to the zebrafish genetic toolbox will enable a variety of experimental strategies requiring precise temporal and spatial control of transgene expression. New TAILOR transgenes employing additional “LSL-R-cDNA-R” cassettes will facilitate a wide variety of experiments requiring sequential and cell type-specific transgene activation and inactivation. As in the case of our *ubb:lox-stop-lox-rox-mCherry-stop-rox-eGFP* allele, recombinase-mediated activation and inactivation of any LSL-Rox-cDNA-Rox cassette is likely to be mosaic. However the incorporation of fluorescent proteins into LSL-Rox-cDNA-Rox alleles, either by in-frame fusion or by 2A peptide-based independent translation [35], will enable the identification of neighboring cells with and without residual transgene expression, allowing such mosaicism to be highly informative. Similarly, the generation of “LSL-RSR-cDNA” alleles, in which expression of a downstream transgene requires both *Cre-* and *Dre-*mediated recombination events, will allow ever more precise temporal and spatial regulation of transgene expression in highly selective cell types characterized by intersectional expression of both *Cre* and *Dre*. *Dre/rox*-based recombination will now also allow independent genetic manipulation of separate transgenes flanked by *lox* and *rox* sites, respectively. For example, *Dre/rox*-based recombination could be used to activate transgene expression in one cell population, with *Cre/lox*-mediated activation of a different transgene occurring in neighboring cells. As capabilities for gene targeting by endonuclease-facilitated homologous recombination [36], [37] become more widespread in zebrafish, the combinatorial application of *Cre/lox* and *Dre/rox* technology will also facilitate temporally regulated and cell type-specific manipulation of endogenous genetic loci.
